# A New Method for Environmental Risk Assessment of Pollutants Based on Multi-Dimensional Risk Factors

**DOI:** 10.3390/toxics10110659

**Published:** 2022-10-30

**Authors:** Le Li, Yuying Dong, Yuting Chen, Jian Jiao, Xuejun Zou

**Affiliations:** College of Environment and Resource, Dalian Minzu University, Dalian 116600, China

**Keywords:** emerging pollutants, environmental risk, life cycle, synthetic risk factor, multidimensional evaluation, systematic assessment

## Abstract

Pollutant discharge causing the deterioration of the watershed environment has seriously threatened human health and ecosystem function. The importance of improving the risk warning system is becoming more and more prominent. Traditional chemical risk assessment methods focused on toxicity and the exposure of pollutants without considering the impact of persistent pollutants in different environmental media. In this study, a new approach was proposed to reflect multi-dimensional evaluation with a synthetic risk factor (SRF) of pollutants. The integrating parameters of SRF include toxicity endpoint values, environmental exposure level, persistent properties, and compartment features. Selected pesticides, perfluorinated compounds, organophosphate esters and endocrine disruptors were analyzed by the proposed and traditional methods. The results showed a higher risk outcome using SRF analysis for PFOS, imazalil, testosterone, androstenedione and bisphenol A, which were different from those obtained by the traditional method, which were consistent with existing risk management. The study demonstrated that the SRF method improved the risk assessment of various pollutants in different environmental media in a more robust fashion, and also provided a more accurate decision basis for ecological environment protection.

## 1. Introduction

With the development of industry and agriculture, more chemicals have been used as medicines, agricultural fertilizers, herbicides and pesticides [1]. Many potentially hazardous substances are released into water and cause water pollution [2]. Although toxic pollutants in environmental water are present at relatively low levels, they can be accumulated in the human body and cause adverse effects [3]. Therefore, it is necessary to assess direct and indirect, short-term and long-term risk, in detail [4]. Environmental risk assessment can help define the possibility of a certain compound to cause severe effects to the environment before these effects are noticed [5]. At present, the most widely used method for the environmental risk assessment of organic pollutants is the risk quotient (RQ) method, which is suitable for the assessment of a single compound [6]. The RQ method is a point estimation method. It cannot provide detailed information about the quality or magnitude of environmental risks, and cannot be used to determine the level of risk [7]. However, increased attention has been paid to a class of chemical substances, namely, emerging pollutants. Evidence has shown that these pollutants exhibit adverse effects at typical ambient concentrations and have not been studied for risk management. These pollutants are often produced from urban, agricultural and wastewater sources. Therefore, risk assessments for individual chemicals may underestimate the risks of contaminants in the actual environment. Persistence coefficient plays an important role in deciding the fate of pollutants in the environment. This study proposed a synthetic risk factor method that incorporated the persistence coefficient of pollutants into the risk quotient calculation. A multi-dimensional risk assessment of pollutants can be carried out, the exposure level of pollutants can be accurately estimated, and corresponding measures can be taken to control the potential risk in time.

The methods of environmental risk assessment are evolving to meet the need for global awareness. The research on environmental risk was originated from the understanding, evaluation and prevention of natural disasters in the 1930s. With the rapid development of industrialization and urban construction in Europe and America, environmental and health problems began to draw attention. At that time, environmental risk studies focused on describing the adverse health effects of human exposure to environmental hazards. In the 1940s, a toxicological identification system was mainly used to analyze the health effects. Quantitative methods have increasingly been used to study the health of populations exposed to different concentrations of pollutants [8]. In the early 1980s, many major environmental pollution incidents occurred in the world [9], which aroused people’s attention and research on environmental health risk assessment, and promoted the gradual standardization of its research. The United States began to adopt the well-known “four-step” method of risk assessment, that is, hazard identification, dose-response relationship assessment, exposure assessment and risk characterization [10]. The method was later adopted by France, the Netherlands, Japan and other countries, as well as some international organizations. In 1992, the United States proposed a more applicable and comprehensive guideline for ecological risk assessment. Based on risk sources, the guideline has taken into account the impact of environmental pollution caused by human life and production on ecosystems [11]. In the mid-1990s, Canada, Britain, Australia and other countries launched environmental risk research work successively, forming a complete environmental risk assessment process [12,13]. With the continuous emergence of various environmental problems, people had paid more attention to the secrecy and uncertainty of future environmental risk changes. It was limited to describe the potential risk in the environment based on individual exposure levels and toxic effects. Later, an “integrated risk assessment” was proposed, which is a risk assessment from human, biological, natural environment and other aspects [14,15]. Nowadays, the environmental risk assessment mainly focuses on the development of risk management and control in the application domain.

Traditional methods for environmental risk assessment, such as the risk quotient (RQ), including environmental exposure level and toxicity endpoint value, were applied as comprehensive parameters to assess the risk of compounds [16,17]. However, environmental risks are also related to the stability and persistence of pollutants in different environmental media. Organic pollutants such as pesticides, microplastics and endocrine disruptors are found in very low concentrations in the environment and are not considered harmful to humans in the short term [18,19]. However, the different effects of these pollutants on the environment and in human health have been demonstrated [20,21]. In addition, uncertainty came from a lack of data on the medium-term and long-term effects and human health of persistent organic pollutants on ecosystems [22]. Further study should be conducted to obtain more POPs exposure data in a wide range of temporal and spatial scales [7]. Thus, in order to evaluate potential risks more comprehensively, we proposed a synthetic risk factor approach by introducing the environmental persistence coefficients of chemicals and the feature of compartments on the basis of the RQ method. The SRF method is based on the assessment of the environmental risk of pollutants in different environmental media. It is not only suitable for water, but also widely suitable for biological phase, soil, air, sediment and other environmental media. Because of the limited data of other environmental media, and the comprehensive data of water body, we focused on the water environment as an example of multi-dimensional risk assessment. Once a chemical risk has been discovered and confirmed, effective measures could be taken for risk management and prevention in time. It is helpful to provide a more comprehensive decision-making basis for ecological environment protection and risk management.

## 2. Materials and Methods

### 2.1. Data Source

The study aimed to conduct risk assessment on pesticide residues, perfluorinated compounds, organophosphate esters and endocrine disruptors detected in the surface waters of the Ebro River, Spain, Tianjin surface water in China, the Bohai Sea, China, and the Xiangjiang River, China. The half-life (T_1/2_) data of pollutants from Chemical Book (https://www.chemicalbook.com/) and other chemical search engines, accessed on 4 September 2022. In the absence of chronic toxicity data, short-term EC_50_ or LC_50_ data should be used to suit for long-term risk assessment. Toxicity data were mainly obtained from the United States EPA ECOTOX database (https://cfpub.epa.gov/ecotox/quick_query.htm), accessed on 4 September 2022. The basic data of residual pesticides, perfluorinated compounds, organophosphates and endocrine disruptors, shown in Table 1, Table 2 and Table 3, were derived from the publications [23,24,25,26]. Our research was focused on the new approach to assess the risk of chemicals. In further study, we will consider the risk of river basins and clarify the water sampling method, location choice and restriction to surface water.

### 2.2. Theory and Methodology

#### 2.2.1. Environmental Persistence Coefficient of the Compound (C)

The measured half-life of a pollutant was associated with the persistence limit value to form the environmental persistence coefficient of the compound:

(1) $$C=T_{\mathrm{CV}}/T_{1/2}$$

where T_1/2_ is the half-life (days) of the pollutant to be measured; T_CV_ is the persistence boundary value (days). T_CV_ is used to describe the stable period of compounds in environmental media. According to the provisions on persistence boundary values in annex D of the Stockholm Convention, “the persistence boundary values were 60 days in the water environment and 180 days in the soil and sediment” [27]. As the persistence boundary value of pollutants, it should be a definite value. Therefore, the 60 (d) was taken as the persistence boundary value to describe the persistence of pollutants in surface water in this paper. The environmental persistence coefficient of chemicals refers to the ratio of the persistence boundary value of chemicals in different environmental media to the half-life of compounds, expressed in C (dimensionless). C is not only related to the persistence boundary value of the compound in different media, but also related to the properties of the compound itself. The smaller the environmental persistence coefficient of the compound, the longer the toxicity of the compound remains in the environment, and the greater the risk to the environment and human health.

#### 2.2.2. Risk Quotient (RQ)

According to EU regulations, in order to assess the risk of pollutants in the aquatic environment, environmental risk assessment calculations for pollutants must be performed. This risk assessment is usually expressed as a risk quotient (RQ). The RQ of an individual pollutant was calculated by dividing the measured environmental concentration (MEC) with the predicted no-effect concentration (PNEC) [7]. The risk quotient were the comprehensive parameters to evaluate the risk of a compound based on the environmental exposure level and toxicity endpoint value. The calculation formula of risk quotient is as follows:

(2) RQ=MEC/PNEC

where MEC is the measured environmental concentration (ng L^−1^); PNEC is the predicted no effect concentration of pollutants (ng L^−1^), which was calculated according to Equations (3) and (4) [28]:

(3) $$PNEC_{acute}=EC_{50}\left(LC_{50}\right)/1000$$

(4) $$PNEC_{chronic}=ChV/100$$

where EC_50_ (LC_50_) represents 50% effective (lethal) concentration, and ChV represents chronic toxic effect.

#### 2.2.3. Synthetical Risk Factor (SRF)

Based on the traditional risk assessment method, the SRF method proposed in this study, introducing the environmental persistence coefficient and compartment characteristics of compounds to evaluate the risk of compounds in the environment. It was a conceptual extension of the RQ method without changing the judgement standard for the environmental risk assessment of chemicals. F(R) is a function of risk with multi-dimensional consideration, resulting in the integrating parameter of SRF. Here, the environmental persistence coefficients of chemicals (C) intruded could reflect the influence of chemical property and compartment characteristics. This can calibrate the results from RQ. The SRF value was calculated according to the following equation:

(5) F(R)=MEC/(PNEC·C)

F(R) represents function relationship of risk, reflecting a certain deterministic relationship between risk and risk factors. Factors influencing risk change include toxicity endpoints values, environmental exposure level, persistent properties and compartment features. 

### 2.3. Scope of Environmental Risk Assessment

Based on the similar logic judgment and evaluation criteria by the RQ method, we could deduce from the risk level of chemicals by SRF. The risk ranking criteria were adopted from the literature [7]: RQ or SRF ≤ 0.01: adverse effects are unlikely to occur and thus can be considered to have negligible hazard; 0.01 < RQ or SRF < 0.1: the minimum risk to the organisms; 0.1 < RQ or SRF < 1: medium risk; and RQ or SRF ≥ 1: high risk.

### 2.4. Procedures

**Figure 1:**
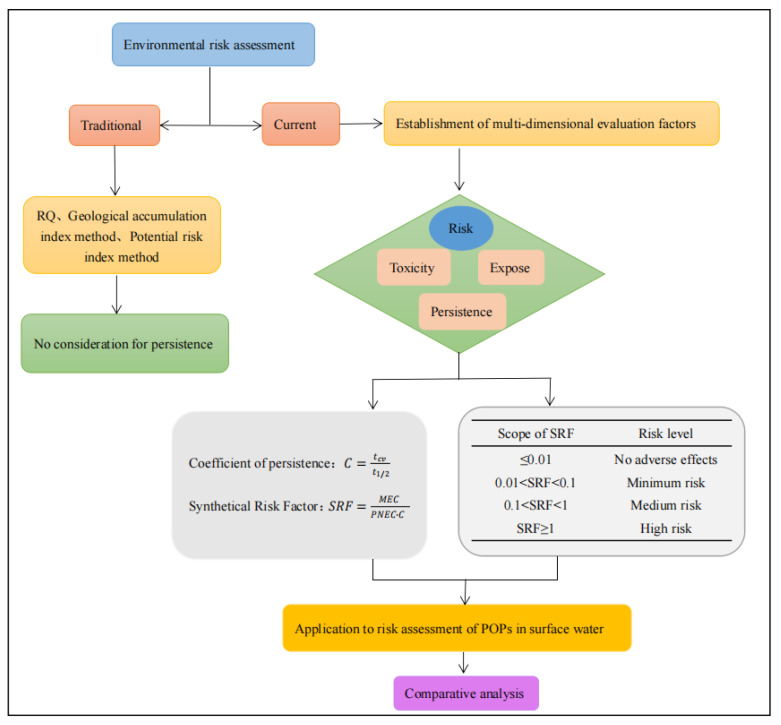
Flow diagram of analyzing environmental risks of pollutants based on multi-dimensional risk factors.

The steps and flow chart for risk assessment using the multi-dimensional evaluation factor are shown in Figure 1:

- Review and collect relevant literature on environmental risk assessment methods; determine the factors affecting environmental risk, and their limitations.
- Establish a comprehensive risk factor function by applying persistence coefficient.
- Apply the function to a risk assessment of emerging pollutants in the selected surface waters and validate accordingly, based on the scope of risk.

### 2.5. Validation Analysis

Four types of target pollutants were selected for the risk assessment; measured environmental concentrations predicted no-effect concentration, and the persistence coefficient was then calculated. The synthetic risk factors were determined and compared with the RQ method to verify the accuracy of the synthetic risk factor method, according to the environmental risk assessment grade.

## 3. Results

### 3.1. Application Domain for Pesticide Residues Based on SRF

**Figure 2:**
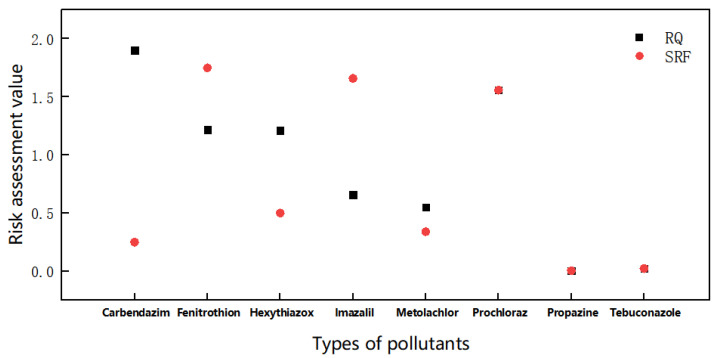
Environmental risk level assessment of residual pesticides using RQ and SRF.

Pesticides have been widely used since the mid-twentieth century for pest control. They are present in a wide range of physicochemical diversity and can be persistent in water, accumulated in sediments, and bio-accumulated in biota, which could cause potential environmental and health issues due to their potential toxicity to non-target organisms [24]. The residual pesticide compounds were sampled from the water of the Ebro River Basin as the target pollutant, followed by a multi-dimensional assessment and analysis of the environmental risks of the pollutants. Basic data of pollutants in river water are shown in Table 1. The comparison of risk levels of pollutants in river basins using both methods are shown in Figure 2.

The results in Figure 2 show that the risk levels for carbendazim (CARB), hexthiazole (HTZ) and imazalil in the pollutants were changed significantly. CARB is a broad-spectrum fungicide and used for foliar spraying, seed treatment and soil treatment. After use, residues may subsequently appear in food, water, soil, or other media, and can also be absorbed by crops and passed along the food chain to humans. Exposure to CARB can lead to downregulation of humoral immune function and the failure of spermatogenesis [29,30]. The risk of CARB is reduced from high to medium by calculating and comparing the two methods. This means that the MEC of CARB is higher than PNEC. However, if the half-life is small, the persistence coefficient will increase. The SRF analysis underestimated the risk assessment value of CARB and could appropriately reduce the risk assessment of CARB. HTZ is an efficient and environmentally friendly acaricide. HTZ is also widely used to control pests of a variety of food crops. According to the European Food Safety Authority (EFSA), it was recently proposed to increase the maximum residue limit of hexythiazox in tea from 0.05 mg/kg to 4 mg/kg, which to a certain extent can reflect the doubts about its environmental risk. The evaluation results showed that the HTZ evaluated by the SRF method was reduced from high risk to medium risk. The longer the half-life of HTZ, the lower the environmental persistence coefficient C, indicated that the pollutants are more persistent to the environment. Even if the risk is reduced, due attention should be paid to risk prevention and control.

The RQ value of imazalil measured by the risk quotient method is 0.66, and the value measured by SRF method is 1.66. According to the scope of risk assessment, the risk of imazalil increased from medium risk to high risk. It showed that imazalil had high persistence in the environment and will cause high risks to the aquatic ecosystem. Pérez-Villanueva et al. [31] studied the occurrence of pesticides in two microcatchments in the Reventazón Basin of North Cartago, Costa Rica, and found they were consistent with our results. Imazalil had a worst-case RQ of 1.1, which was high risk. These fungicides, like imazalil, have been used in some agribusinesses at concentrations up to 0.6–2 g L^−1^ to control fungal infections during fruit and seed storage [32]. As a result, high amounts of imazalil residues were released during the washing step, producing a high volume of contaminated wastewater [33,34,35]. Imazalil is also recommended for use as a fungicide by the Ministry of Agriculture in China. Although its toxicity is low, due to the high emission and stronger environmental sustainability, the monitoring of its environmental emission should be further strengthened. In addition, the European Union’s Food Safety Authority (EFSA) recommend that the potential environmental risk of azole drugs should be under close vigilance after reviewing the maximum residue limits (MRLS) for imazole in certain foods in on 30 October 2018.

As expected, after evaluating the worst-case scenario, imazalil exhibited a high risk to aquatic organisms, while the presence of carbendazim and hexythiazox posed a moderate risk to aquatic organisms. The presence of pesticide residues in the aquatic environment, particularly at high concentrations, may cause detrimental effects on aquatic organisms and eventually human beings. The SRF value depended on the toxicity endpoint values, environmental exposure level, persistence properties and compartment features. In pesticide risk management, it is necessary to comprehensively consider the features, production, use, environmental residue and other factors of residual pesticides. Environmental risks are assessed at a higher level and prioritized.

### 3.2. Application Domain for PFCs and OPEs Based on SRF

**Figure 3:**
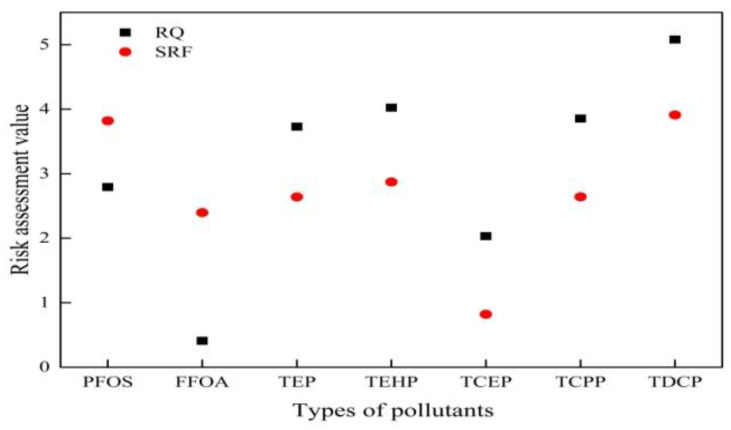
Environmental risk level assessment of perfluorinated compounds and organophosphate esters using RQ and SRF.

In this study, the surface water of Tianjin city and the Bohai Sea were sampled for risk assessment; target pollutants included perfluorinated compounds and organophosphate esters. Basic data such as chemical formula and the half-life of pollutants are shown in Table 2. The evaluation results are shown in Figure 3. Since the difference between the risk assessment values are large and cannot be seen clearly in the figure, the risk assessment values on the y-axis in the graph are logarithmic.

Perfluorinated compounds (PFCs) have been recognized as emerging global pollutants and have attracted scientific and political attention worldwide. To date, PFCs have been found to be released into the environment and biological matrices through the use of PFC-containing products, or through the degradation of their precursors. Due to their bioaccumulation and multiple toxicities, PFCs are persistent in the environment and widely present in wildlife and humans [36,37]. Their presence in the environment poses a risk to ecosystems. As the half-life of PFOS increased, its environmental persistence coefficient decreased. Based on both the RQ and the SRF methods, it was found that the risk value of PFOS was less than 1, and its risk level changed from low to medium. Therefore, PFOS in surface water will persist in the environment for a long time, which will cause a certain degree of pollution to the environment. The risk level of PFOA did not change and was considered a very low risk. Huang et al. [38] evaluated perfluoroalkyl substances (PFASs) in surface water and sediment samples from the inland river basin in the Longgang District, and the RQ values were all less than 1. The results were consistent with our study; however, the risk of PFCs contamination in surface water to ecosystems should draw more attention because of their bioaccumulation [39]. The government and environmental departments should formulate environmental quality standards for perfluorinated compounds in different environmental media, so as to provide an assessment basis for the accurate assessment of ecological risks of perfluorinated compounds in environmental media. It is necessary to strengthen the construction of the environmental management system for perfluorinated compounds, monitor and supervise the emergence of perfluorinated compounds substitutes in real time, and the early prevention and control of their risks.

Organophosphate esters (OPEs) are widely used in plastics, textiles, building materials, lubricants, electronics and coatings, due to their excellent physicochemical properties and high cost performance [40]. Although incidents of environmental contamination from OPE are rarely reported, they are ubiquitous in the environment as re-emerging contaminants considered to be potential health concerns, and research on the toxic effects of these chemicals is increasing. Bioaccumulation data and risk assessments of OPEs in aquatic organisms such as fish, algae and snails have been reported [41]. The SRF values of TEP, TEHP, TCEP, TCPP and TDCP in the organophosphate, in the Figure 3, were all less than 1, indicating that there was no risk for aquatic organisms in the watershed. Cristale et al. [42] evaluated the risks of 10 OPEs in the Piracicaba River Basin (Brazil). The RQ of most OPEs was lower than 1 to 4 orders of magnitude, which means that the risk to aquatic organisms was negligible. This conclusion was consistent with our research.

### 3.3. Application Domain for Endocrine Disruptors Based on SRF

**Figure 4:**
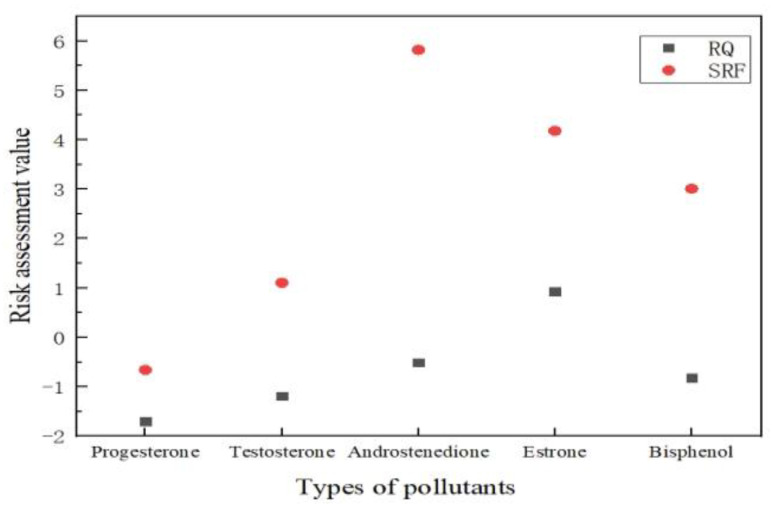
Environmental risk level assessment of endocrine disruptors using RQ and SRF.

Endocrine disruptors (EDs) are compounds that cause great environmental problems. EDs are a large group of substances of natural or anthropogenic origin that interfere with an organism’s endocrine system. They interact with estrogen receptors to enhance or inhibit the normal function of the hormone, potentially leading to adverse effects such as sterility and species extinction in aquatic organisms [43]. Although such pollutants are usually present in the environment in low concentrations, some of them are toxic per liter concentrations [44]. Their adverse effects leading to certain cancers and other non-communicable diseases, such as diabetes, and adverse effects on aquatic life populations, have been reported [45]. In this study, endocrine disruptors in the surface water of Xiang Jiang River were used as target pollutants to evaluate their environmental risks. Related pollutant data are shown in Table 3. The RQ and SRF methods were used to calculate the risk value of each pollutant of endocrine disruptors in the Xiang Jiang River. The comparison results are shown in Figure 4. The logarithm of the risk assessment values of the y-axis in Figure 4 is taken.

The lack of regulated monitoring has resulted in increasing concentrations of micropollutants in the environment and increased public concern about the presence of endocrine disrupting compounds in surface waters. In order to remove these compounds, appropriate identification and evaluation methods need to be employed. The risk values were significantly changed for progesterone, testosterone, androstenedione and bisphenol A, from low to high risk, using the SRF method. Chronic exposure to testosterone at lower concentrations can cause endocrine disruption in aquatic animals [46]. In addition, the environmental pollutant levels of estrogen may contribute to breast cancer in women, prostate cancer in men, and reproductive system abnormalities in men [47]. Androstenedione belongs to steroidal androgens. It mainly comes from the excrement and urine of livestock and poultry, as well as the discharge of wastewater from paper mills and urban sewage treatment plants. The continuous discharge of pollution sources leads to the male phenomenon of fish in some areas, which has a strong negative impact on species richness and ecological balance, and raises the environmental risk.

Bisphenol A (BPA) is a well-known endocrine disrupting compound commonly found in industrial wastewater and wastewater treatment plants [48]. The widespread use of BPA in the plastics industry has led to its widespread distribution in the environment and to inevitable human exposure to the substance through dietary and non-dietary sources [49,50]. Previous studies have shown that BPA can be detected in dust, surface water, industrial sewage, sediment and soil. BPA can interact with estrogen and nuclear receptors to varying degrees, interfering with their natural expression, thereby acting as endocrine disruptors [51]. There is good evidence that BPA at 1–10 μg mL^−1^ is acutely toxic to freshwater and marine species, and this disturbance can adversely affect reproductive and metabolic functions. This has prompted strict EU regulations on the use of BPA in some industrial products, leading to the widespread use of its structural and functional analogs in manufacturing, such as bisphenol AF (BPAF) [52,53,54]. Combining the persistence level and the exposure effect, the risk assessment level is high, so the release of BPA in the water environment and its further identification are still worthy of attention. The pollution route and concentration level of EDs in different environmental media were systematically understood. The risk assessment system and efficient and applicable risk control technology of EDCs were studied. The priority endocrine disrupting substances should be identified as early as possible through instrumental analysis.

## 4. Conclusions

As risks are gradually recognized by people, risk assessment theories are constantly enriched and developed, and we should pay close attention to pollutants with potential environmental risks. Traditional chemical risk assessment methods are mostly based on the toxicity endpoint value and the environmental exposure level of pollutants, aiming at the environmental pollution that has occurred. The accuracy of environmental risk prediction for some new pollutants that are not included in routine environmental monitoring but may enter the environment and cause known or potential negative ecological or health effects needs to be improved.

In this study, a multi-dimensional environmental risk assessment method combined with a pollutant stabilization period was proposed. The diagnostic results indicated that the assessment method is more accurate to determine the environmental risk level of unknown risk pollutants, and data were consistent with those from existing control measures. Compared with the existing methods based on the theoretical basis, the SRF method is superior in the identification of toxic substances by introducing the persistence properties of chemicals and the feature of compartments. The SRF method can be more comprehensively applied to discover new environmental pollutants from different dimensions, improve the risk prediction performance of new pollutants, and provide better environmental early warning references. It has helped protect human health, biology and other environmental components. Therefore, the application of persistence coefficient in the environmental risk assessment method improved the risk assessment. Under risk estimates that incorporate a persistence coefficient, the compound’s properties, production, usage, environmental residues and other factors should be considered in risk assessment and management. Given that compounds existed in water bodies and aquatic organisms for a longer period of time, appropriate measures should be taken to reduce the input of toxic substances into rivers and strengthen the supervision of illegal discharge of industrial sewage. Administrative departments, the WHO and water management authorities should timely change the management protocols for water quality and public health. With further research ongoing, a deep understanding and intensive linkage could be helpful for us to make a more accurate identification model about the actual risk level of pollutants in different environmental media. The obtained data by SRF includes more information to disclose the pollutant risk in different environmental media, which could provide support to predict and find chemical risk in time. Since samples were collected from a few selected areas in this study, for future research, the sampling sites can be appropriately expanded to study the risks of different new pollutants, which have broad application prospects.

## Figures and Tables

**Table 1:** Basic data of pesticide residues in the Ebro River.

Compound	CAS	Chemical Formula	Half-Life (d)	Persistence Boundary Value (d)	C	MEC (ng/L)	PNEC (ng/L)
Carbendazim	10605-21-7	C_9_H_9_N_3_O_2_	8	60	7.5	2.78	1.5
Fenitrothion	122-14-5	C_9_H_12_NO_5_PS	86.1	60	0.70	0.11	0.09
Hexythiazox	78587-05-0	C_17_H_21_ClN_2_O_2_S	24.6	60	2.44	7.41	6.1
Imazalil	35554-44-0	C_14_H_14_Cl_2_N_2_O	151	60	0.40	61.01	92
Metolachlor	51218-45-2	C_15_H_22_ClNO_2_	39	60	1.54	0.55	1
Prochloraz	67747-09-5	C_15_H_16_Cl_3_N_3_O_2_	60	60	1	15.59	18
Propazine	139-40-2	C_9_H_16_ClN_5_	90	60	0.67	0.14	40
Tebuconazole	107534-96-3	C_16_H_22_ClN_3_O	62	60	0.97	2.36	100

**Table 2:** Basic data of perfluorinated compounds in surface water of Tianjin, China, and organophosphate esters in the Bohai Sea, China.

Compound		Abbr.	CAS	Chemical Formula	Half-Life (d)	Persistence Boundary Value (d)	C	MEC (ng/L)	PNEC (ng/L)
Perfluorinated compounds	Perfluorrooctane sulphonate	PFOS	1763-23-1	C_8_HF_17_O_3_S	1.48 × 10^4^	60	0.0041	1.61	1000
	Perfluorooctanoic acid	PFOA	335-67-1	C_8_HO_2_F_15_	1.58 × 10^3^	60	0.038	15.1	100,000
Organophosphate esters	Triethyl phosphate	TEP	78-40-0	C_6_H_15_O_4_P	4.90	60	12.24	1.683	9.0 × 10^5^
	Tri(2-ethylhexyl) phosphate	TEHP	78-42-2	C_24_H_51_O_4_P	4.23	60	14.18	47.4	5.0 × 10^5^
	Tri(2-chloroethyl) phosphate	TCEP	115-96-8	C_6_H_12_Cl_3_O_4_P	3.68	60	16.30	473.79	5.1 × 10^4^
	Tris(1-chloro-2-propyl) phosphate	TCPP	13674-84-5	C_9_H_18_Cl_3_O_4_P	3.68	60	16.30	6.3	4.5 × 10^4^
	Tris(1,3-dichloro-2-propyl) phosphate	TDCP	13674-87-8	C_12_H_15_Cl_6_O_4_P	4.08	60	14.71	3.249	3.9 × 10^4^

**Table 3:** Basic data of endocrine disruptors in surface water of Xiangjiang River, China.

Compound	CAS	Chemical Formula	Half-Life (d)	Persistence Boundary Value (d)	C	MEC (ng/L)	PNEC (ng/L)
Progesterone	57-83-0	C_21_H_30_O_2_	666.66	60	0.09	8.1	415
Testosterone	58-22-0	C_19_H_28_O_2_	1.17 × 10^4^	60	5.1 × 10^−3^	6.5	100
Androstenedione	1963-5-8	C_19_H_26_O_2_	1.12 × 10^3^	60	5.4 × 10^−2^	4.4	14
Estrone	53-16-7	C_18_H_22_O_2_	1.06 × 10^5^	60	5.7 × 10^−4^	51.33	6
Bisphenol A	1980-5-7	C_15_H_16_O_2_	4.02 × 10^6^	60	1.5 × 10^−5^	30.9	2000

## Data Availability

The data presented in this article are available on request from the corresponding authors.
